# Differential Effects of the Catechol-O-Methyltransferase Val158Met Genotype on the Cognitive Function of Schizophrenia Patients and Healthy Japanese Individuals

**DOI:** 10.1371/journal.pone.0076763

**Published:** 2013-11-25

**Authors:** Shoko Tsuchimine, Norio Yasui-Furukori, Ayako Kaneda, Sunao Kaneko

**Affiliations:** Department of Neuropsychiatry, Graduate School of Medicine, Hirosaki University, Hirosaki, Japan; Oregon Health & Science University, United States of America

## Abstract

**Background:**

The functional polymorphism Val158Met in the catechol-O-methyltransferase (COMT) gene has been associated with differences in prefrontal cognitive functions in patients with schizophrenia and healthy individuals. Several studies have indicated that the Met allele is associated with better performance on measures of cognitive function. We investigated whether the COMT Val158Met genotype was associated with cognitive function in 149 healthy controls and 118 patients with schizophrenia.

**Methods:**

Cognitive function, including verbal memory, working memory, motor speed, attention, executive function and verbal fluency, was assessed by the Brief Assessment of Cognition in Schizophrenia (BACS-J). We employed a one-way analysis of variance (ANOVA) and a multiple regression analysis to determine the associations between the COMT Val158Met genotype and the BACS-J measurements.

**Results:**

The one-way ANOVA revealed a significant difference in the scores on the Tower of London, a measure of executive function, between the different Val158Met genotypes in the healthy controls (*p* = 0.023), and a post-hoc analysis showed significant differences between the scores on the Tower of London in the *val/val* genotype group (18.6 ± 2.4) compared to the other two groups (17.6 ± 2.7 for *val/met* and 17.1 ± 3.2 for *met/met*; *p* = 0.027 and *p* = 0.024, respectively). Multiple regression analyses revealed that executive function was significantly correlated with the Val158Met genotype (*p* = 0.003). However, no evidence was found for an effect of the COMT on any cognitive domains of the BACS-J in the patients with schizophrenia.

**Conclusion:**

These data support the hypothesis that the COMT Val158Met genotype maintains an optimal level of dopamine activity. Further studies should be performed that include a larger sample size and include patients on and off medication, as these patients would help to confirm our findings.

## Introduction

The enzyme catechol-O-methyltransferase (COMT) metabolizes the neurotransmitters dopamine, adrenalin and noradrenalin, and COMT is the main component in the dopamine metabolic pathway [1,2]. The enzymatic activity of COMT is altered by a guanine (G) to adenine (A) single nucleotide polymorphism (SNP) (known as Val158Met or rs4680) in the sequence of the gene. This SNP translates into a valine (Val) to methionine (Met) amino acid change in codon 158, which causes a three- to four-fold decrease in the molecular thermostability of this enzyme [3]. The alleles appear to be codominant, and the heterozygous genotype (Val158Met) is associated with an intermediate level of COMT activity [4,5]. High COMT activity leads to a hypodopaminergic state; low COMT activity has the opposite effect. 

The activity of COMT in the prefrontal cortex (PFC) affects dopamine-mediated cognitive function in schizophrenic patients and healthy individuals [6]. This high level of activity has been hypothesized to result in the poorer performance of frontally mediated cognitive tasks because of the lower dopamine levels [7]. The majority of the investigations of the relationship between the COMT Val158Met genotype and cognitive function have focused on schizophrenic patients, in whom the *val* allele has typically been associated with impairments in prefrontal abilities [8-12]. However, other investigators have not supported these findings [13-15]. In healthy individuals, although some researchers have observed an association between the lower-activity *met* allele of the COMT gene and better performance on measures of prefrontal executive functioning [16-18] and working memory [9], several other researchers have found no association between the COMT Val158Met genotype and cognitive performance [14,19,20]. However, there have been few studies in this field with healthy Japanese individuals.

 Recently, Kaneda et al. [21] reported that the Brief Assessment of Cognition in Schizophrenia, Japanese-language version (BACS-J), was superior in the clinical evaluation of the cognitive function of schizophrenic patients. The authors reported that the time required for testing with the BACS was only approximately 30 min, with minimal extra time for scoring and training. The BACS is simple and easy, and unlike the Wechsler Adult Intelligences Scale-Revised (WAIS-R), the BACS has the advantage of being able to investigate several cognitive functions as primary measures, including verbal memory, working memory, motor speed, attention, executive function and verbal fluency [21]. 

 In this study, we investigated whether the COMT Val158Met polymorphism affected cognitive function assessed by the BACS-J in healthy controls and in patients with schizophrenia. Our results showed a relatively weak association between executive function within the six cognitive domains and COMT Val158Met polymorphism in the healthy controls but not in the patients with schizophrenia.

## Methods

### 1: Subjects

 Participants were 118 stably maintained outpatients with chronic schizophrenia and 149 healthy controls (Table 1). The demographic results of the healthy controls and the patients with schizophrenia showed significant differences in gender, age, and education. Because matching for all the demographic factors led to a drastic reduction in the number of subjects in each genotype, we did not match for these factors. Instead, we gave priority to matching the estimated premorbid IQ for the three genotypes in each diagnostic group. All the participants were native Japanese speakers and were right-handed (>70), according to Oldfield’s Edinburgh Inventory [22].

**Table 1:** Clinical characteristics of study groups.

	Patients with Schizophrenia					Healthy controls					*Group difference
Val158Met SNP (rs4680)	*val/val*	*val/met*	*met/met*	*F*	*p*	*val/val*	*val/met*	*met/met*	*F*	*p*	t-test
Variable	(n = 56)	(n = 47)	(n = 15)			(n = 68)	(n = 62)	(n = 19)			*p*
Gender (male/female)	27/29	25/22	8/7	0.144	n.s.	52/16	46/16	15/4	0.101	n.s.	**0.02**
Age	42.3 (14.6)	39.1 (14.4)	30.1 (7.5)	4.312	**0.016**	36.4 (11.2)	36.4 (10.3)	38.9 (14.1)	0.417	n.s.	**0.01**
Education, years	12.6 (2.1)	13.5 (5.7)	13.5 (6.6)	0.440	n.s.	14.0 (2.1)	14.0 (2.2)	14.2 (2.3)	0.048	n.s.	**0.03**
Estimated premorbid IQ	97.9 (11.7)	96.4 (10.1)	95.2 (9.8)	0.445	n.s.	101.7 (8.7)	100.3 (8.1)	104.5 (8.4)	1.177	n.s.	n.s.
Age at onset, years	28.0 (8.7)	24.5 (8.8)	24.2 (6.7)	1.713	n.s.	NA	NA	NA			
Duration of illness, years	14.9 (11.9)	10.9 (9.5)	6.5 (5.3)	3.395	**0.039**	NA	NA	NA			
Chlorpromazine equivalent dose, mg/day	536.6 (421.3)	532.4 (369.6)	872.6 (998.3)	1.028	n.s.	NA	NA	NA			

* *T*-test was used for testing group difference in patients and controls distribution. Otherwise, ANOVA was used.

IQ, Intelligence Quotientt; NA, not applicable.

 The patients were recruited from outpatients at the University of Hirosaki Hospital and two affiliated hospitals. The diagnosis of schizophrenia was made through the Structured Clinical Interview for DSM-IV Axis I Disorders (SCID) [23] by an experienced psychiatrist (N.YF.). To screen the healthy controls, the SCID non-patient edition (SCID-NP) was used. Premorbid IQs were estimated using the Japanese version of the National Adult Reading Test [24] (Table 1).

 The exclusion criteria for both groups were neurological illness, traumatic brain injury with any known cognitive impairment, a history of electroconvulsive therapy, and alcohol/substance abuse or addiction. An additional exclusion criterion for the healthy control group was a personal history of psychiatric disease or a family history of an axis I disorder in any of their first-degree relatives.

 All participants provided written informed consent to participate in the study after a complete description of the procedure. The care takers or guardians consented on the behalf of participants whose capacity to consent was compromised provided written informed consent to participate in the study. The study protocol was approved by the Ethics Committee of the Hirosaki University School of Medicine.

 After the participants provided consent, the cognitive tests were administered, and the blood samples were taken. All participants were financially reimbursed. 

### 2: Genotyping

 DNA was extracted from 5 ml of whole blood by manual extraction, using the QIAamp DNA Blood Maxi Kit (QIAGEN, Tokyo, Japan).

 The COMT Val158Met polymorphism was detected using a real time 5’-nucleosidase (TaqMan) reaction employing the following reagents: forward primer 5’- CCCAGCGGATGGTGGAT-3, reverse primer 5’-CAGGCATGCACACCTTGTC-3’, VIC-labeled probe, 5’-TTCGCTGGCATGAAG-3’, and FAM-labeled probe 5’-TCGCTGGCGTGAAG-3’. The PCR mixture contained a total volume of 25 μl, consisting of 20-50 ng genomic DNA, 40 × primer/probe mixture, and 2 × TaqMan Universal PCR Master Mix (Applied Biosystems Inc., Foster City, CA, USA). The PCR cycling reactions were performed in 96-well PCR plates in an ABI PRISM 7000 Sequence Detection System (Applied Biosystems) and consisted of an initial denaturation step of 10 min at 95°C followed by 40 cycles of 95°C for 15 sec and 60°C for 1 min. The results were analyzed using ABI PRISM 7000 SDS version 1.0.1 software (Applied Biosystems).

### 3: Cognitive Assessment

 The participants were evaluated with the BACS-J. The BACS-J includes brief assessments of verbal memory, working memory, motor speed, verbal fluency, attention, and executive function. The following is a description of the six subtests of the BACS-J: List Learning (verbal memory), Digit Sequencing Task (working memory), Token Motor Task (motor speed), Verbal Fluency (processing speed), Symbol Coding (attention and processing speed), and Tower of London (executive function/reasoning and problem solving).

### 4: Data Analysis

 A one-way analysis of variance (ANOVA) was performed to examine the genotype groups. Gender, age, education, BACS-J subtests scores and the composite score were the dependent variables, and the COMT Val158Met genotype was the independent factor. As a post-hoc comparison between the groups, the Fisher’s least significant difference (LSD) test was applied.

 A multiple linear regression analysis was performed to determine which factors (gender, age, education, and COMT Val158Met genotype) correlated with the BACS-J measures and the composite score. 

 The data were analyzed using SPSS PC software for Windows, version 15.0. Statistical significance was defined as *p* < 0.05.

## Results

The genotypic distributions of three genotypes in the COMT gene were as follows: healthy controls, *val/val*, n = 68 (45.6%), *val/met*, n = 62 (41.6%), and *met/met*, n = 19 (12.8%); and patients with schizophrenia, *val/val*, n = 68 (47.5%), *val/met*, n = 62 (39.8%), and *met/met*, n = 19 (12.7%). The distributions of the three genotypes were not significantly different from those expected according to the Hardy-Weinberg equilibrium in either diagnostic group. The demographic characteristics of the genotype groups are shown in Table 1. There were significant differences between the genotypes for age and duration of illness in the patients with schizophrenia (*p* = 0.016 and *p* = 0.036, respectively). The three genotypic groups did not differ significantly in gender, age, year of education, and premorbid IQ in the healthy controls (Table 1). Also, gender, education, premorbid IQ, age at onset, and dose of medication were not significantly different between the genotype groups in healthy controls (Table 1).

In the healthy controls, a one-way ANOVA revealed a significant association between the COMT Val158Met genotype and scores on the Tower of London (*F* = 3.855, *p* = 0.023). The LSD test revealed that individuals with the *val/val* genotype had a significantly higher score than did those individuals with either the *val/met* or the *met/met* genotype (*p* = 0.027 and *p* = 0.024, respectively). There were no associations for the other BACS-J measures (Table 2). Multiple regression models that included gender, age and education showed that there was a significant correlation between the COMT Val158Met genotype and the Tower of London (*R* ^2^ = 0.236, β = 0.225, *t* = 3.061, *p* = 0.003), but the COMT Val158Met genotype was not correlated with the other five BACS-J measures or the composite score (Table 3). We also showed a correlation between age and the Verbal Memory (beta = -0.432, *p* < 0.001), Digit Sequencing Task (beta = -0.363, *p* < 0.001), Verbal Fluency (beta = -0.380, *p* < 0.001), Symbol Coding (beta = -0.478, *p* < 0.001), Tower of London (beta = -0.324, *p* < 0.001), and composite score (beta = -0.491, *p* < 0.001). In addition, gender was independently associated with the Tower of London (beta = 0.172, *p* = 0.029). Education level was also associated with the Verbal Memory (beta = 0.220, *p* = 0.005), Token Motor Task (beta = 0.230, *p* = 0.009), Symbol Coding (beta = 0.165, *p* = 0.029), and composite score (beta = 0.249, *p* = 0.001). 

**Table 2:** Mean performance of patients and controls for each measurement of the COMT Val158Met genotype.

	Patients with Schizophrenia					Healthy controls				
Val158Met SNP (rs4680)	*val/val*	*val/met*	*met/met*	*F*	*p*	*val/val*	*val/met*	*met/met*	*F*	*p*
Variable	(n = 56)	(n = 47)	(n = 15)			(n = 68)	(n = 62)	(n = 19)		
Verbal Memory	27.6 (12.9)	31.4 (11.2)	28.5 (10.9)	1.304	n.s.	46.0 (9.4)	47.2 (10.7)	45.5 (9.0)	0.329	n.s.
Digit Sequencing Task (working memory)	15.1 (5.0)	15.4 (4.5)	16.2 (4.8)	0.354	n.s.	20.7 (4.2)	19.5 (4.3)	19.2 (4.5)	1.796	n.s.
Token Motor Task (moter speed)	67.4 (14.6)	69.6 (21.9)	72.4 (20.6)	0.487	n.s.	92.7 (9.3)	92.1 (9.3)	90.7 (11.2)	0.327	n.s.
Verval Fluency (processing speed)	34.1 (11.4)	33.1 (11.0)	34.5 (11.4)	0.128	n.s.	45.0 (9.9)	45.0 (8.6)	47.9 (12.2)	0.755	n.s.
Symbol Coding (attention and processing speed)	45.4 (15.0)	48.4 (14.0)	49.1 (11.0)	0.777	n.s.	67.5 (12.3)	67.7 (9.7)	66.0 (16.2)	0.164	n.s.
Tower of London (executive function/reasoning and problem solving)	13.9 (4.7)	15.2 (4.9)	15.9 (4.5)	1.569	n.s.	18.6 (2.4)	17.6 (2.7)	17.1 (3.2)	3.855	**0.023**
Composite Score	203.4 (50.2)	213.3 (52.2)	216.6 (49.9)	0.662	n.s.	290.5 (33.0)	289.0 (32.1)	286.3 (43.2)	0.117	n.s.

Data indicate means±SD.

COMT, catechol-O-methyltransferase; BACS-J, Brief Assessment of Cognition in Schizophrenia, Japanese-language version; SNP, single nucleotide polymorphism.

**Table 3:** Factors that influenced scores of the BACS-J measuremnts and the composite scores of multiple regression analysis.

		Patients with Schizophrenia					Healthy control			
BACS-J measures	Variable	*beta*	*t*	*p*	*R^2^*		*beta*	*t*	*p*	*R^2^*
Tower of London	Gender	-0.069	-0.672	0.504	0.335		0.172	2.208	**0.029**	0.236
(executive function/reasoning and problem solving)	Age	-0.450	-3.977	**<0.001**			-0.324	-4.078	**< 0.001**	
	Education	0.275	2.715	**0.008**			0.028	0.348	0.728	
	COMT Val158Met	-0.066	-0.592	0.556			0.225	3.061	**0.003**	

COMT, catechol-O-methyltransferase; BACS-J, Brief Assessment of Cognition in Schizophrenia, Japanese-language version.

In the patients with schizophrenia, the ANOVA did not reveal a significant difference for any of the cognitive domains measured by the BACS-J (Table 2). Multiple regression analysis revealed that there was a correlation between age and the Verbal Memory (beta = -0.370, *p* < 0.005), Digit Sequencing Task (beta = -0.486, *p* < 0.001), Verbal Fluency (beta = -0.339, *p* = 0.004), Symbol Coding (beta = -0.683, *p* < 0.001), Tower of London (beta = -0.450, *p* < 0.001), and composite score (beta = -0.511, *p* < 0.001). In addition, gender was independently associated with the Token Motor Task (beta = -0.231, *p* = 0.045). Education level was also associated with the Digit Sequencing Task (beta = 0.243, *p* < 0.022), Verbal Memory (beta = 0.388, *p* < 0.001), Symbol Coding (beta = 0.203, *p* = 0.026), Token Motor Task (beta = 0.230, *p* = 0.009), Tower of London (beta = 0.275, *p* = 0.008), and composite score (beta = 0.307, *p* = 0.002).

## Discussion

 The result of our study indicated that executive function, as measured by the Tower of London subtest in the BACS-J, was associated with the COMT Val158Met polymorphism in healthy controls. Individuals with the *val/val* genotype performed significantly better than did those individuals with the *val/met* or *met/met* genotype. However, no evidence was found of an effect of the COMT on any cognitive domains of the BACS-J in patients with schizophrenia. These different results between healthy control subjects and patients with schizophrenia may have been due to the decline of dopamine transmission, the affinity for the dopamine receptors that are affected by antipsychotic drugs [25], and the illness itself [26]. The effect of the COMT genotype on prefrontal dopamine availability is probably minimal compared with the massive dopamine-blocking effect of antipsychotic drugs. Therefore, it was assumed that because the cognitive function in patients with schizophrenia had highly inter-individual variability, a significant difference was not found in patients with schizophrenia. These findings suggest that the activity of COMT may influence cognitive function in healthy controls. 

A previously proposed hypothesis for the opposite effects of the COMT Val158Met genotypes on cognition is that there may be an optimum level of dopamine signaling in a healthy individual’s brain and that healthy individuals with the *val/val* genotype and those individuals with schizophrenia start off on different points on an inverted-U curve model and thus are affected differently by the COMT Val158Met genotype [27]. This study implied that the dopamine levels of healthy individuals with the COMT *met* variant are excessive compared to those individuals with the *val* variant and occurred well into the downward right slope of the inverted-U curve. That is, there is a possibility that the healthy individuals with the high activity *val/val* genotype had a better performance on the cognitive tasks than did those individuals with the low activity *met/met* genotype because the intrinsic dopamine level in the healthy individuals was higher than it was in the patients with schizophrenia. Although no significant differences between the genotype groups on any cognitive functions of BACS-J were found in the patients with schizophrenia, our results are in-line with the inverted-U curve (for example, in executive function *val/val*: 13.9, *val/met*: 15.2, *met/met*: 15.9 for patients with schizophrenia; *val/val*: 18.6, *val/met*: 17.6, *met/met*: 17.1 for healthy controls). Therefore, our results may have supported an inverted-U curve model.

 Previous studies have not identified consistent associations between cognitive function and the COMT Val158Met polymorphism. Some studies [7,9,16,28] and a meta-analysis [11] have found an association between the lower-activity *met* allele of the COMT gene and better performance on cognitive measures in healthy volunteers. These results are inconsistent with our observation showing no difference in composite scores on BACS-J between the COMT Val158Met polymorphisms. There have been a few studies supporting our results, in which the presence of the *val* allele had a positive impact on cognitive function in healthy volunteers [29]. reported that those individuals who were homozygous for the *val* allele performed better on an assessment of cognitive function than did individuals in either the *val/met* or the *met/met* genotype groups. In addition, a recent study that simultaneously used healthy subjects and patients with schizophrenia demonstrated that the effect of the COMT Val158Met genotype on the cognitive function of healthy controls was opposite to the effect in patients with schizophrenia [30]. More studies have reported that the *val* genotypes of the COMT Val158Met polymorphism have a negative effect on cognitive tasks that engage executive function in patients with schizophrenia [7,9,31,32]. Because schizophrenia is hallmarked by relative decreases in prefrontal dopamine activity [33], patients with schizophrenia who carry the *val* allele may be exposed to a risk for the prefrontal degradations of dopamine, which has been associated with decrements in cognitive function [34,35]. Because the prefrontal dopamine activities of healthy individuals are constantly kept at an adequate level, their prefrontal dopamine signaling may be less vulnerable to the negative regulations by the *val* alleles. Therefore, the *val* variants of the COMT gene may have a different effect on cognitive function in healthy individuals than they do in patients with schizophrenia.

 The COMT Val158Met genotype may contribute to differences in working memory and executive function [34,35]. In our results, healthy individuals with the *val/val* genotype had a non-significant trend toward higher scores on the Digit Sequencing Task, which measured working memory, than did those individuals with the *val/met* and *met/met* genotypes (*val/val*: 20.7, *val/met*: 19.5, *met/met*: 19.2, *p* = 0.092). This trend was likely non-significant because our sample size was small, and we used a method of cognitive measurement that was different from previous studies [9,12]. In addition, the BACS was developed as an assessment of cognitive function in schizophrenic patients and was not sensitive enough to detect a small difference in working memory in healthy individuals. We found that the heterozygotes group (*val/met*) of healthy controls showed an intermediate executive function score. This result was consistent with previous studies supporting an allelic codominance at the COMT Val158Met locus [4,5], which have confirmed that the polymorphism has a highly relevant effect in modulating the activity of COMT, although other polymorphisms in the gene may also contribute [36].

 Our study has several limitations that must be considered in interpreting our results. First, our sample size was relatively small for a genetic study. Scores on the Tower of London were very similar, likely because of the small sample size. If a more rigorous statistical analysis was applied after the one-way ANOVA, then the significant difference disappeared (*p* > 0.017 after Bonferroni correction). Therefore, there is a possibility that the association between the COMT Val158Met genotype and executive function was relatively weak. Second, the lack of an association between the COMT Val158Met genotype and prefrontal tasks may have occurred because we did not employ measures such as the Wisconsin Sorting Card Test (WSCT) or the N-back task in the current investigation [9,11,12,36]. Third, our sample was composed of high functioning relatively well-educated medical staff, and the literature suggests that neuropsychological measures may have limited sensitivity in such individuals due to the increased potential for cognitive and neuropsychological reserves. 

## Conclusion

 We observed a relatively weak association between the COMT Val158Met genotype and executive function in healthy controls. Individuals with the *val/val* genotype performed significantly better than did those individuals with the *val/met* and *met/met* genotype, which is in contrast with previous studies. These data may support the hypothesis that the COMT Val158Met genotype maintains an optimal level of the dopamine activity. Further studies should be performed that include a larger sample size and include patients on and off medication, as these patients would help to confirm our findings. 
